# Supplementary material for: Rapid Uniformity Analysis of Fully Printed SWCNT-Based Thin Film Transistor Arrays via Roll-to-Roll Gravure Process
Source: Nanomaterials (Basel). 2023 Feb 1;13(3):590. doi: 10.3390/nano13030590 (PMC9920362; doi:10.3390/nano13030590)
Supplement: Supplementary file 1 [file nanomaterials-13-00590-s001.zip › nanomaterials-2177012-supplementary.pdf]

nanomaterials

Supplementary Materials

Rapid Uniformity Analysis of Fully Printed SWCNT-based Thin Film Transistor Arrays via Roll-to-Roll Gravure Process

Yunhyok Choi [1†], Younsu Jung [2†], Reem Song [1], Jinhwa Park [2], Sajjan Parajuli [3], Sagar Shrestha[2] and Gyoujin Cho [2,3,*] and Byung-Sung Kim [1,*]

[1] Department of Semiconductor Systems Engineering, College of Information and Communication Engineering, Sungkyunkwan University, Suwon-si, 16419, Rep. of Korea
[2] Department of Biophysics, Institute of Quantum Biophysics, Research Engineering Center for R2R printed Flexible Computer, Sungkyunkwan University, Suwon-si, 16419, Rep. of Korea
[3] Department of Intelligent Precision Healthcare Convergence, Institute of Quantum Biophysics, Research Engineering Center for R2R printed Flexible Computer, Sungkyunkwan University, Suwon-si, 16419, Rep. of Korea
* Correspondence: gcho1004@skku.edu & bskimice@skku.edu
† These authors contributed equally to this work

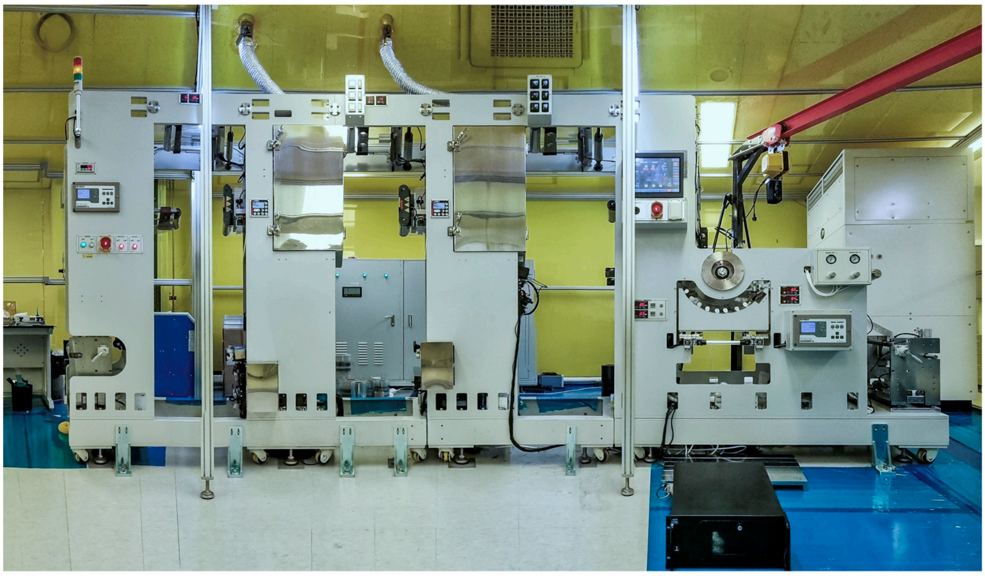

Figure S1. R2R gravure printing system with two printing unit.

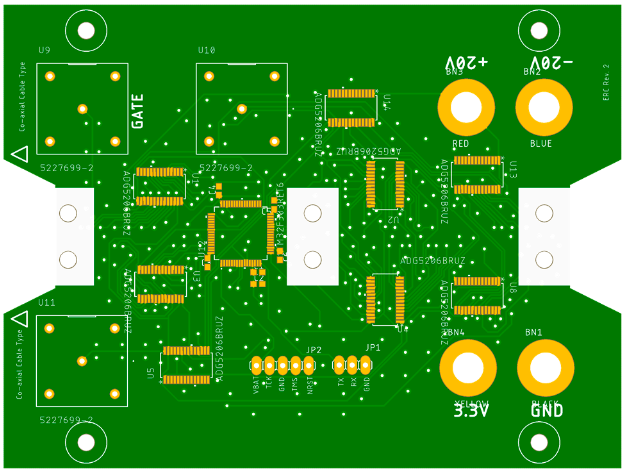

Figure S2. Circuit layout of a probing instrument dedicated to quickly characterizing R2R-printed TFT array.

Table 1. Each average leakage current and resistance between probes for a SWCNT-TFTs

Column	1	2	3	4	5	6	7	8	9	10
Resistance (Ω)	375.2	374.6	375.1	374.1	374.2	372.9	372.6	371.4	375.8	374.9
Leakage Current (pA)	519.8	775.3	123.5	113.6	118.5	111.1	98.8	114.8	98.8	104.9
Column	11	12	13	14	15	16	17	18	19	20
Resistance (Ω)	375.4	374.6	374.4	373.2	372.7	371.4	374.0	373.2	373.7	372.7
Leakage Current (pA)	82.7	108.6	106.2	103.7	104.9	82.7	119.8	104.9	80.2	114.8
Column	21	22	23	24	25	26	27	28	29	30
Resistance (Ω)	372.8	371.8	371.6	370.5	374.7	373.6	373.9	373.1	373.0	372.0
Leakage Current (pA)	95.1	56.8	79.0	92.6	92.6	95.1	504.9	72.8	79.0	75.3
Column	31	32	33	34	35	36	37	38	Average Value	
Resistance (Ω)	371.7	370.6	380.3	379.5	380.2	378.7	380.5	379.8	374.3	
Leakage Current (pA)	80.2	49.4	71.6	17.3	49.4	50.6	695.1	58.0	144.8	

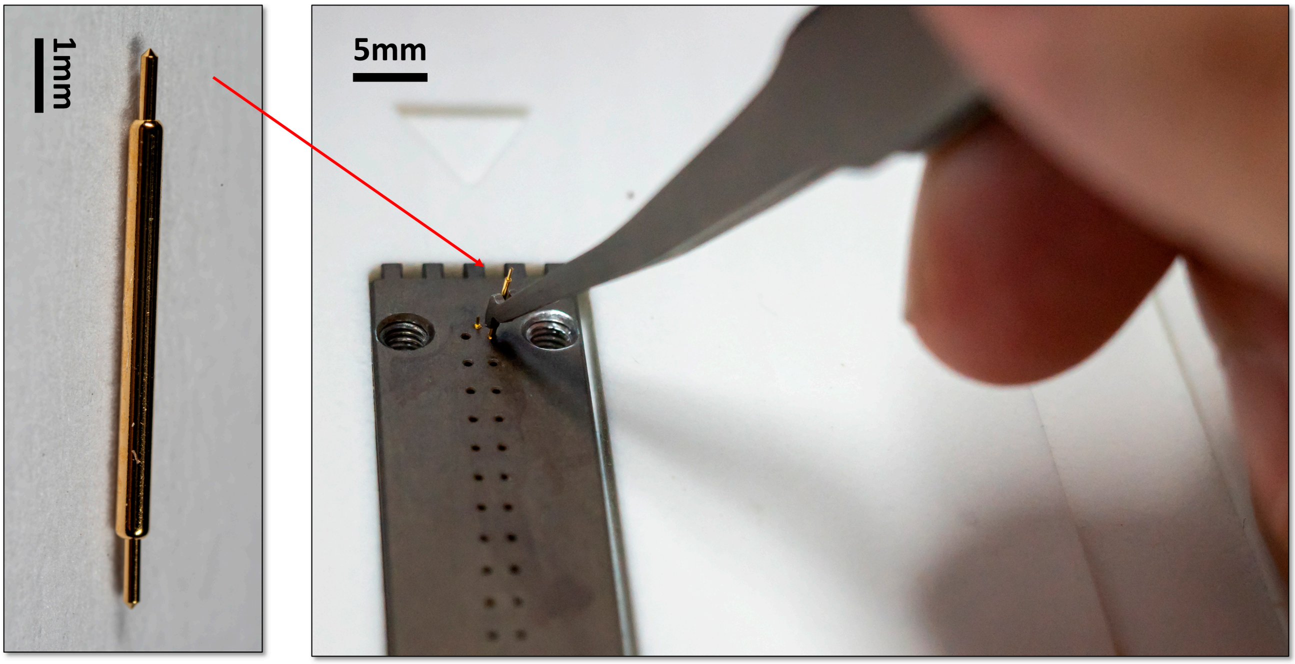

Figure S3. Excellent repairability of pogo-pin type probes.

(a) **(b)**

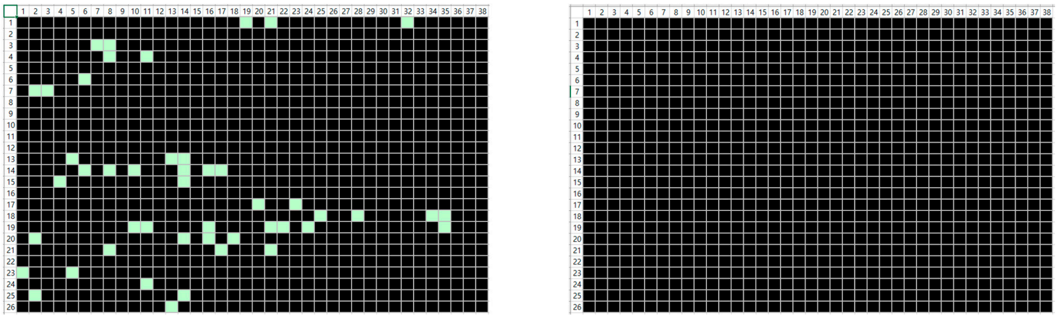

Figure S4. Statistical maps generated from PICR2R-TFT measurements of the 26 x 38 SWCNT-TFT arrays with a printing speed of 30 mm/s containing different channel lengths : (a) 15 µm and (b) 25 µm (light box :"pass", dark box :"fail").
